# Mortality in *Escherichia coli* bloodstream infections: a multinational population-based cohort study

**DOI:** 10.1186/s12879-021-06326-x

**Published:** 2021-06-25

**Authors:** Melissa C. MacKinnon, Scott A. McEwen, David L. Pearl, Outi Lyytikäinen, Gunnar Jacobsson, Peter Collignon, Daniel B. Gregson, Louis Valiquette, Kevin B. Laupland

**Affiliations:** 1 Department of Population Medicine, University of Guelph, Guelph, Ontario Canada; 2 Department of Health Security, National Institute for Health and Welfare, Helsinki, Finland; 3 Department of Infectious Diseases, Skaraborg Hospital, Skövde, Sweden; 4 CARe - Center for Antibiotic Resistance Research, Institute of Biomedicine, University of Gothenburg, Gothenburg, Sweden; 5 Department of Infectious Disease and Microbiology, The Canberra Hospital, Garran, Australian Capital Territory Australia; 6 Medical School, Australian National University, Acton, Australian Capital Territory Australia; 7 Departments of Medicine, and Pathology and Laboratory Medicine, University of Calgary, Calgary, Alberta Canada; 8 Alberta Health Services, Calgary Zone, Calgary, Alberta Canada; 9 Department of Microbiology-Infectious Diseases, Université de Sherbrooke, Sherbrooke, QC Canada; 10 Department of Medicine, Royal Inland Hospital, Kamloops, British Columbia Canada; 11 Department of Intensive Care Medicine, Royal Brisbane and Women’s Hospital, Brisbane, Queensland Australia; 12 Faculty of Health, Queensland University of Technology, Brisbane, Queensland Australia

**Keywords:** Population-based, Bloodstream infection, Bacteremia, *Escherichia coli*, Mortality, Mortality rate, Case fatality

## Abstract

**Background:**

*Escherichia coli* is the most common cause of bloodstream infections (BSIs) and mortality is an important aspect of burden of disease. Using a multinational population-based cohort of *E. coli* BSIs, our objectives were to evaluate 30-day case fatality risk and mortality rate, and determine factors associated with each.

**Methods:**

During 2014–2018, we identified 30-day deaths from all incident *E. coli* BSIs from surveillance nationally in Finland, and regionally in Sweden (Skaraborg) and Canada (Calgary, Sherbrooke, western interior). We used a multivariable logistic regression model to estimate factors associated with 30-day case fatality risk. The explanatory variables considered for inclusion were year (2014–2018), region (five areas), age (< 70-years-old, ≥70-years-old), sex (female, male), third-generation cephalosporin (3GC) resistance (susceptible, resistant), and location of onset (community-onset, hospital-onset). The European Union 28-country 2018 population was used to directly age and sex standardize mortality rates. We used a multivariable Poisson model to estimate factors associated with mortality rate, and year, region, age and sex were considered for inclusion.

**Results:**

From 38.7 million person-years of surveillance, we identified 2961 30-day deaths in 30,923 incident *E. coli* BSIs. The overall 30-day case fatality risk was 9.6% (2961/30923). Calgary, Skaraborg, and western interior had significantly increased odds of 30-day mortality compared to Finland. Hospital-onset and 3GC-resistant *E. coli* BSIs had significantly increased odds of mortality compared to community-onset and 3GC-susceptible. The significant association between age and odds of mortality varied with sex, and contrasts were used to interpret this interaction relationship. The overall standardized 30-day mortality rate was 8.5 deaths/100,000 person-years. Sherbrooke had a significantly lower 30-day mortality rate compared to Finland. Patients that were either ≥70-years-old or male both experienced significantly higher mortality rates than those < 70-years-old or female.

**Conclusions:**

In our study populations, region, age, and sex were significantly associated with both 30-day case fatality risk and mortality rate. Additionally, 3GC resistance and location of onset were significantly associated with 30-day case fatality risk. *Escherichia coli* BSIs caused a considerable burden of disease from 30-day mortality. When analyzing population-based mortality data, it is important to explore mortality through two lenses, mortality rate and case fatality risk.

**Supplementary Information:**

The online version contains supplementary material available at 10.1186/s12879-021-06326-x.

## Background

*Escherichia coli* bloodstream infections (BSIs) are associated with an important burden of disease from mortality, because *E. coli* is the most common cause of bacterial BSIs [1–3]. In order to better understand the contribution of mortality to the overall burden of disease from *E. coli* BSIs, population-based studies are required [4]. Two general approaches for analyzing mortality data from population-based studies include evaluation of mortality rates and case fatality risks, which provide distinct yet complementary results [3, 5]. Mortality rates (number of deaths per population in a given time period) provide insight into the disease burden and case fatality risks (number of deaths per total number of cases) are markers of disease severity [5]. Appropriate comparison of mortality rates and case fatality risks from different bacterial causes of BSIs can help facilitate prioritization of funding, research, and surveillance.

A small number of published population-based studies have reported mortality data for *E. coli* BSIs, however, they did not all use the same definition of mortality [1, 6–11]. All-cause mortality definitions used included in-hospital mortality, in-hospital 7-day mortality, and 30-day mortality [1, 6–11]. The reported in-hospital case fatality risks for *E. coli* BSIs ranged from 8.9–11% [6, 7, 9]. One study reported an in-hospital 7-day case fatality risk of 5% for *E. coli* BSIs [8] and three studies reported 30-day case fatality risks between 8 and 18.2% [1, 10, 11]. Mortality rates for *E. coli* BSIs were only reported by three studies [3] (in-hospital mortality rate of 2.9 deaths/100,000 person-years, and 30-day mortality rates of 7 and 10.3 deaths/100,000 person-years) [6, 10, 11].

Antimicrobial-resistant *E. coli* BSIs have been associated with increased mortality and three population-based studies explored this association [6, 9, 11]. Ciprofloxacin-resistant [6, 11] and extended-spectrum β-lactamase (ESBL) producing [9] *E. coli* BSIs were associated with higher in-hospital mortality. Three previous population-based studies demonstrated significantly increased mortality in patients with hospital-onset *E. coli* BSIs (in-hospital mortality [6, 7] and 30-day mortality [11]). Multidrug resistant *E. coli* BSIs*,* which can include resistance to carbapenems and are associated with hospital-onset and healthcare-associated BSIs, can have significant impacts on the ability to successfully treat infections and prevent mortality [12–16]. Previous population-based studies reported mortality data for *E. coli* BSIs that were diagnosed prior to 2015, which highlights the opportunity to report more recent mortality data. We are not aware of a previously published multinational population-based study exploring mortality in *E. coli* BSIs.

Using data from a multinational population-based cohort of *E. coli* BSIs, we had two main objectives for our study: first, to evaluate the 30-day case fatality risk and factors associated with case fatality risk; and second, to evaluate the 30-day mortality rate and factors associated with mortality rate.

## Methods

### Surveillance populations and study protocol

We enrolled five areas from the International Bacteremia Surveillance Collaborative in three countries and two continents for this population-based cohort study [17]. The surveillance areas (2018 population) included: Calgary Health Region, Canada (1.7 million), country of Finland (5.5 million), Sherbrooke Region, Canada, (166,000), Skaraborg County Health Region, Sweden (267,000), and western interior area of British Columbia, Canada (191,000). Previous publications outline each area’s population and surveillance methodology [17–19]; Skaraborg now has two hospitals instead of four as previously published. Each area’s surveillance database captures at least 99% of all positive blood cultures from residents [17, 19]. The 5-year study (01/01/2014–12/31/2018) included all incident *E. coli* BSIs from area residents and we defined incident as the first *E. coli* isolate cultured from blood per patient per running year (at least 1 year of time elapsed between *E. coli* BSIs). Depending on the area, we retrieved data from electronic medical records or national registers (infectious disease, population, and hospital discharge registers), including: 30-day and 7-day all-cause mortality, year of culture, patient’s sex and age category (< 1 year, 1–9 years, then deciles until ≥90 years), location of onset (hospital-onset or community-onset), and susceptibility to third-generation cephalosporins (3GC). All-cause 30-day mortality was defined as death due to any reason within 30-days after the positive *E. coli* blood culture. We categorized BSIs as hospital-onset if the first positive blood culture was obtained at least 48 h after hospital admission or within 48 h of discharge, otherwise they were categorized as community-onset [20]. Each area used their own protocols for susceptibility testing. Using standardized templates, areas collected and compiled their own data. The study was approved by the Interior Health Research Ethics Board and University of Guelph Research Ethics Board, and individual informed consent waivers were granted (2013–14-052-I and 2018–10-050, respectively). As appropriate, local and / or national ethics requirements were adhered to by all other areas. All research methods were completed in accordance with the Declaration of Helsinki. Analyses related to incidence rates and antimicrobial resistance for *E. coli* BSIs in this study are documented in a separate manuscript [21].

### Statistical analyses for case fatality risks

We performed statistical analyses in Stata 15.1 [22]. Based on *E. coli* BSI patients with 30-day mortality, we calculated proportions to summarize dichotomous variables (sex, location of onset, and 3GC resistance) and to summarize age, we determined the age category that contained the median 30-day death. Using univariable logistic regression, an odds ratio (OR) was estimated to compare the odds of a BSI being hospital-onset in 3GC-resistant (R) and 3GC-susceptible (S) *E. coli* BSI patients with 30-day mortality. We calculated 30-day case fatality risk by dividing the number of 30-day deaths by the number of incident *E. coli* BSIs. Thirty-day case fatality risks were calculated for each level of the dichotomous variables: age (< 70-years-old and ≥ 70-years-old), sex, location of onset, and 3GC resistance.

We used a logistic regression model to determine factors significantly associated with 30-day mortality in *E. coli* BSIs. The six categorical explanatory variables considered for inclusion in the logistic regression model included: year (2014 through 2018), region (five study areas), age (< 70-years-old and ≥ 70-years-old), sex (female and male), 3GC resistance (S and R), and location of onset (community-onset and hospital-onset). First, we completed univariable analysis and checked for correlation between explanatory variables using a Phi coefficient (ρ ≥ |0.8| was used as the threshold value) followed by building the multivariable logistic regression model starting with all of the explanatory variables. Three interaction effects were considered for inclusion in the final multivariable model: year and region, age and sex, and 3GC resistance and location of onset. In order to remain in the final multivariable model, variables had to meet at least one of the following criteria: be statistically significant (α = 0.05), be part of a significant interaction term, or be a confounding variable (based on > 20% change in another variable’s coefficient and meeting causal criteria) [23]. We assessed the final multivariable model for goodness-of-fit using a Hosmer-Lemeshow goodness-of-fit test [23]. We assessed Pearson standardized residuals, leverage, and influence statistics (delta-beta, delta-chi^2^, and delta-deviance) for each covariate pattern or observation [23]. If interaction terms were included in the final multivariable model, contrasts were used to interpret these relationships. Odds ratios (OR) were reported with 95% CI.

### Statistical analyses for mortality rates

We calculated 30-day mortality rate by dividing the number of 30-day deaths by the population during the study period (from individual area census data). Direct age and sex standardization of the 30-day mortality rate to the European Union 28-country (EU28) 2018 population was performed to allow comparison of mortality rates between different regions and different years [22, 24]. The process above was repeated for 3GC-R and 3GC-S 30-day mortality data.

To identify factors significantly associated with *E. coli* BSI 30-day mortality rates, we used a Poisson regression model [22, 23]. The four categorical explanatory variables considered for inclusion in the Poisson regression model, included: year (2014 through 2018), region (five study areas), age (< 70-years-old and ≥ 70-years-old), and sex (female and male). We performed univariable analysis and tested for correlation between explanatory variables (as described for the logistic regression model) before placing all explanatory variables in the multivariable Poisson regression model. Interaction effects between year and region, and age and sex were considered for inclusion in the final multivariable model. In order to remain in the final multivariable model, variables had to meet at least one of the following criteria: be statistically significant (α = 0.05), be part of a significant interaction term, or be a confounding variable (based on > 20% change in another variable’s coefficient and meeting causal criteria) [23]. We assessed the fit of the final multivariable Poisson regression model by assessing the normality of Anscombe residuals and the significance of the overdispersion parameter when the model was re-fit as a negative binomial regression model (null-hypothesis that alpha = 0; *p* > 0.05 indicating the Poisson model fits the data) [23]. We assessed residuals (Pearson and deviance), leverage, and an influence statistic (Cook’s distance) for each covariate pattern or observation [23]. Incidence rate ratios (IRR) were reported with 95% confidence intervals (CI).

## Results

**Fig. 1:**
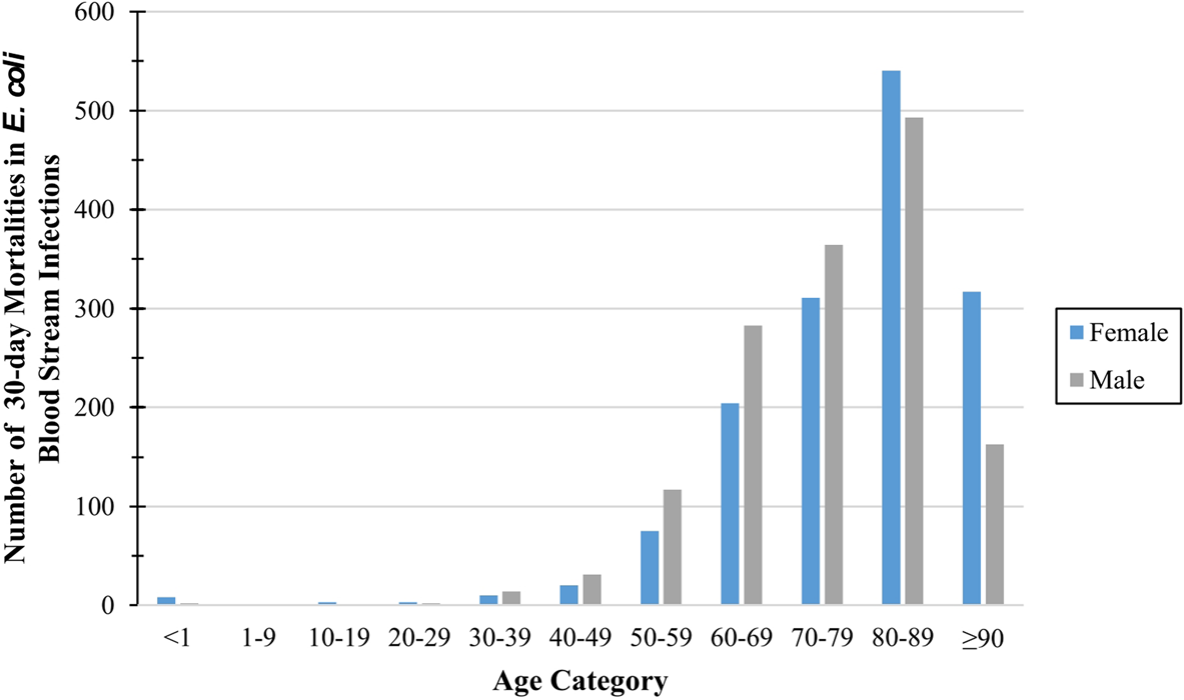
Number of 30-day deaths in *E. coli* bloodstream infection patients by age category and sex

During our 5-year study, there were 2961 30-day deaths in 30,923 incident *E. coli* BSIs, which we identified from 38.7 million person-years of surveillance. There was a left skewed age distribution, and the median age range of patients that experienced 30-day mortality was 80–89-years-old (Fig. 1). Thirty-day deaths in patients with *E. coli* BSIs were evenly distributed between the sexes (female 50.3%, 1490/2961). We found 11.4% (336/2961) of the *E. coli* BSIs that resulted in 30-day deaths were resistant to 3GC and this ranged from 3.5% (5/142) in Skaraborg to 24.2% (109/450) in Calgary. Most of the *E. coli* BSIs that resulted in 30-day deaths were community-onset *E. coli* BSIs (67.1%, 1987/2961); this proportion was lowest in Finland (65.4%, 1472/2252) and highest in Skaraborg (78.2%, 111/142). When considering only the *E. coli* BSI patients with 30-day mortality, those with 3GC-R *E. coli* BSIs were at greater odds of being hospital-onset compared to 3GC-S (OR:1.51, 95%CI:1.20–1.91, *p* < 0.001). Regional data for 3GC resistance and location of onset is available in Supplementary table 1, Additional file 1.

The overall 30-day case fatality risk was 9.6% (2961/30923) and ranged from 7.2% (43/596) in Sherbrooke to 12.8% (74/578) in western interior (see Supplementary table 2, Additional file 1). Patients ≥70-years-old experienced an 11.4% (2188/19277) 30-day case fatality risk compared to 6.6% (773/11646) in those < 70-years-old. Thirty-day case fatality risk was higher in males (11.7%, 1470/12598) compared to females (8.1%, 1490/18325). Patients with 3GC-R *E. coli* BSIs had a 14.1% 30-day case fatality risk (336/2376), which was higher than those with 3GC-S *E. coli* BSIs (9.2%, 2625/28547). Thirty-day case fatality risk in hospital-onset *E. coli* BSIs was 17.7% (974/5506) compared to 7.8% (1987/25417) in community-onset *E. coli* BSIs. Of the 30-day deaths, 50.4% (1493/2961) occurred within 7-days of BSI diagnosis, which ranged from 47.2% (1063/2252) in Finland to 68.9% (51/74) in western interior.

With univariable logistic regression analysis, region, location of onset, 3GC resistance, sex, and age were all significantly associated with 30-day mortality in *E. coli* BSIs (see Supplementary table 3, Additional file 1). Our multivariable logistic regression model for 30-day mortality included region, location of onset, 3GC resistance and an interaction between age and sex (Table 1). Calgary, Skaraborg, and western interior had higher odds of 30-day mortality compared to Finland (Table 1). Compared to community-onset *E. coli* BSIs, patients with hospital-onset *E. coli* BSIs had higher odds of 30-day mortality (Table 1). Patients with *E. coli* BSIs resistant to 3GC had higher odds of dying within 30 days compared to those with 3GC-S *E. coli* BSIs (Table 1). Regarding the interaction between sex and age, when males were compared to either females of the same age category or younger females, males had higher odds of 30-day mortality (Table 2). When older females were compared to either younger females or males, the older females had higher odds of 30-day mortality (Table 2). When the two age categories of males were compared, older males had increased odds of 30-day mortality (Table 2).

**Table 1:** Multivariable logistic regression model results estimating associations between explanatory variables and *E. coli* BSI 30-day mortality^a^

Variable	aOR	95% CI	***p***-value
**Region**			< 0.001^b^
Finland	1.00	referent	
Calgary	1.52	1.36–1.71	< 0.001
Sherbrooke	0.87	0.64–1.20	0.403
Skaraborg	1.31	1.09–1.57	0.004
Western interior	1.69	1.31–2.18	< 0.001
**Location of Onset**			
Community-onset	1.00	referent	
Hospital-onset	2.44	2.25–2.66	< 0.001
**3GC-R**			
Susceptible	1.00	referent	
Resistant	1.37	1.20–1.55	< 0.001
**Sex**			
Female	1.00	referent	
Male	1.77^c^	1.52–2.05	< 0.001
**Age Category**			
< 70-years-old	1.00	referent	
≥ 70-years-old	2.21^c^	1.95–2.52	< 0.001
**Interaction - Sex and Age**			
Male and ≥ 70	0.73^c^	0.62–0.87	< 0.001

*Abbreviations*: *BSI* Bloodstream infection, *aOR* Adjusted odds ratio, *CI* Confidence interval

^a^Model fits the data based on Hosmer-Lemeshow goodness-of-fit test (*p* = 0.653)

^b^Overall *p*-value for variable estimated using a likelihood ratio test

^c^Exponentiated coefficients are not true aOR due to interaction term – see contrasts in Table 2

**Table 2:** Results for contrasts examining interactions between sex and age based on multivariable logistic regression model^a^

Contrast Statement	aOR	95% CI	***p***-value
Males < 70 compared to females < 70	1.77	1.52–2.05	< 0.001
Males ≥70 compared to females ≥70	1.30	1.18–1.42	< 0.001
Males ≥70 compared to females < 70	2.87	2.52–3.28	< 0.001
Females ≥70 compared to females < 70	2.21	1.95–2.52	< 0.001
Males ≥70 compared to males < 70	1.62	1.44–1.83	< 0.001
Females ≥70 compared to males < 70	1.25	1.11–1.41	< 0.001

*Abbreviations*: *aOR* Adjusted odds ratio, *CI* Confidence interval

^a^Multivariable logistic regression model estimating the associations between the explanatory variables (region, location of onset, third-generation cephalosporin resistance, sex, and age) and 30-day mortality in *E. coli* bloodstream infection (Table 1)

**Fig. 2:**
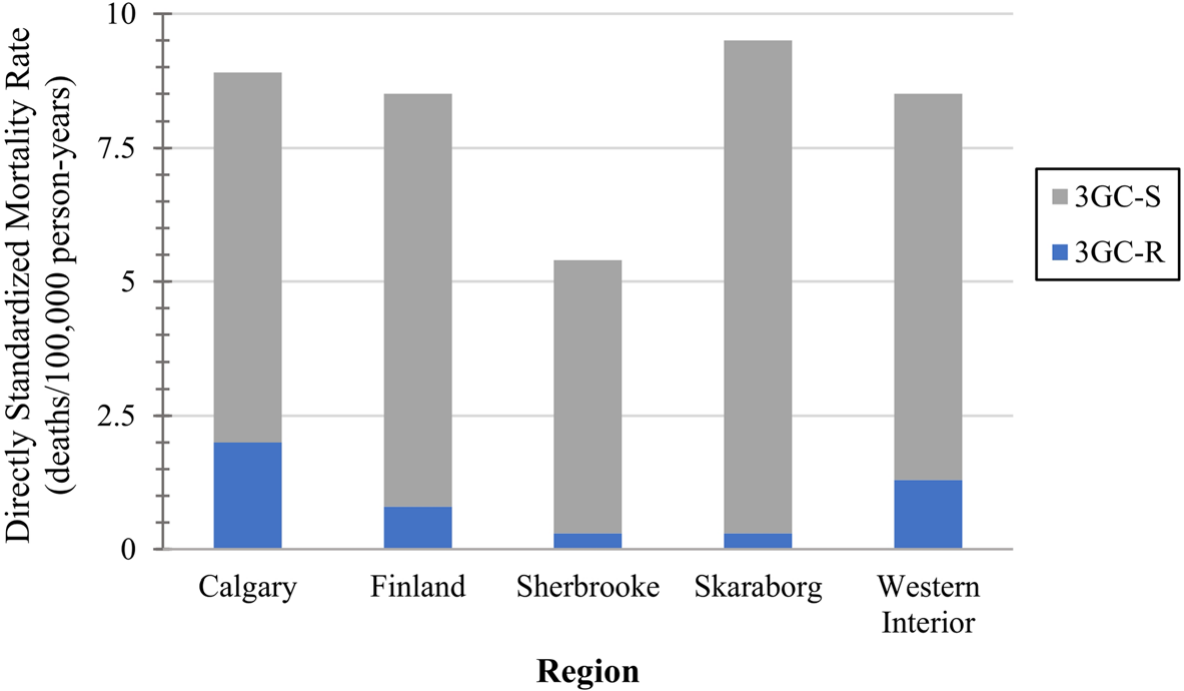
Directly age and sex standardized *E. coli* bloodstream infection 30-day mortality rates by area^a^ Abbreviations: 3GC-R – Third-generation cephalosporin-resistant; 3GC-S – Third-generation cephalosporin-susceptible. ^a^Standard population – EU28 2018 population

**Fig. 3:**
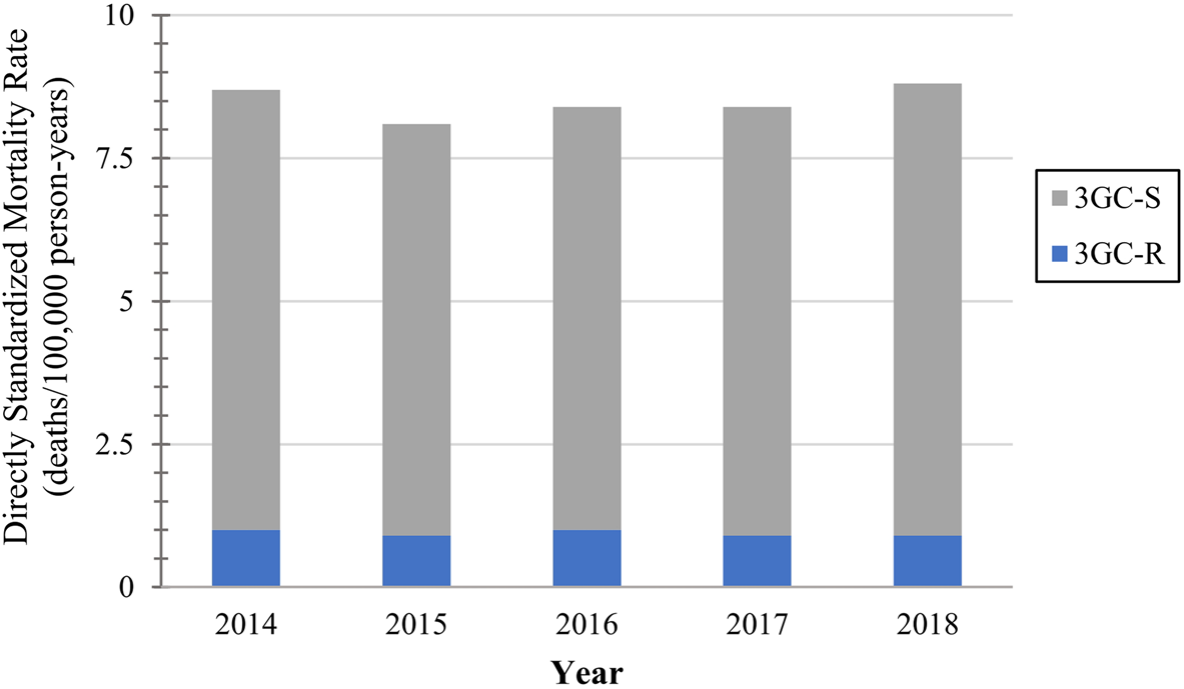
Directly age and sex standardized *E. coli* bloodstream infection 30-day mortality rates by year^a^. Abbreviations: 3GC-R – Third-generation cephalosporin-resistant; 3GC-S – Third-generation cephalosporin-susceptible. ^a^Standard population – EU28 2018 population

The overall crude 30-day mortality rate was 7.7 deaths/100,000 person-years. Supplementary table 2, Additional file 1 contains regional mortality data, and crude rates. The overall directly age and sex standardized mortality rate was 8.5 deaths/100,000 person-years, which was lowest in Sherbrooke, 5.4 deaths/100,000 person-years, and highest in Skaraborg, 9.6 deaths/100,000 person-years (Fig. 2). The directly standardized 3GC-R mortality rate was 0.9 deaths/100,000 person-years, which ranged from 0.3 to 2.0 deaths/100,000 person-years (Sherbrooke and Skaraborg, and Calgary, respectively) (Fig. 2). The directly standardized overall and 3GC-R mortality rates were relatively stable over the five-year study (Fig. 3). Regional and annual standardized overall, 3GC-R, and 3GC-S mortality rates are presented in Supplementary table 4, Additional file 1.

With univariable Poisson regression analysis, region, and age were significantly associated with *E. coli* BSI 30-day mortality rate (see Supplementary table 5, Additional file 1). Our multivariable Poisson regression model for 30-day mortality rate included region, sex, and age (Table 3). Sherbrooke had a significantly lower 30-day mortality rate compared to Finland (Table 3). Compared to female *E. coli* BSI patients, male patients had a significantly higher 30-day mortality rate (Table 3). The 30-day mortality rate was significantly higher in patients that were ≥ 70-years-old compared to those < 70-years-old (Table 3).

**Table 3:** Multivariable Poisson regression model results estimating associations between explanatory variables and *E. coli* BSI 30-day mortality rate^a^

Variable	aIRR	95% CI	***p***-value
**Region**			0.035^b^
Finland	1.00	referent	
Calgary	0.99	0.89–1.09	0.796
Sherbrooke	0.67	0.49–0.90	0.009
Skaraborg	1.14	0.96–1.35	0.138
Western interior	0.99	0.78–1.24	0.913
**Sex**			
Female	1.00	referent	
Male	1.26	1.17–1.35	< 0.001
**Age Category**			
< 70-years-old	1.00	referent	
≥ 70-years-old	20.35	18.73–22.11	< 0.001

*Abbreviations*: *BSI* Bloodstream infection, *aIRR* Adjusted incidence rate ratio, *CI* Confidence interval

^a^Model fits the data based on normally distributed Anscombe residuals and lack of significant overdispersion (overdispersion parameter in negative binomial model [*p* = 0.302])

^b^Overall *p*-value for variable estimated using a likelihood ratio test

## Discussion

Our population-based study provides important and novel insights into mortality in *E. coli* BSIs using data from five areas in three countries on two continents over a recent five-year period. Due to the population-based design of our cohort study, we were able to move beyond evaluation of case fatality risks to explore mortality rates. We used multivariable regression models to control for confounding variables while investigating risk factors for *E. coli* BSI mortality rate and case fatality risk.

Mortality is an important component of burden of disease in *E. coli* BSIs and was examined in only a limited number of previous population-based studies [1, 6–11]. We can cautiously compare our results to those from previously published population-based studies, but it is important to note that the studies are from different time periods and populations, and the studies used a variety of mortality definitions. In-hospital mortality has been shown to be a biased measure of mortality at a general population-level [25] and is likely influenced by factors associated with health system and healthcare delivery. Regional-population based studies from Canberra (Australia; 2000–2004; in-hospital 7-day mortality), Calgary (Canada; 2000–2006; in-hospital), mid-Norway (Norway; 2002–2013; 30-day), Auckland (New Zealand; 2005–2011; in-hospital), and two Thai provinces (Thailand; 2008–2014; in-hospital) reported case fatality risks of 5, 11, 8.6, 9, and 8.9%, respectively [6–10]. National surveillance in Finland (2004–2007) and England (07/2011–06/2012) reported 30-day case fatality risks of 8 and 18.2%, respectively [1, 11]. Our overall 30-day case fatality risk of 9.6% is similar to most of the case fatality risks reported in previous population-based studies. Non-population-based *E. coli* BSI studies reported extremely variable 30-day case fatality risks ranging from 2.8 to 37.5% [12–14, 26–38]; our 30-day case fatality risk is in the lower end of the range. However, the patients in the non-population-based studies are typically from highly selected populations including tertiary care centres, which consequently, provide minimal insight into the severity of *E. coli* BSIs at a general population-level.

We identified a 30-day crude mortality rate of 7.7 deaths/100,000 person-years, which was comparable to the 30-day crude mortality rate of 7 deaths/100,000 person-years from the mid-Norway study [10]. However, our rate was higher than that reported in a study from Calgary (in-hospital crude mortality rate, 2.9 deaths/100,000 person-years), and calculated for a study from Finland (30-day crude mortality rate, 3.5 deaths/100,000 person-years) [1, 6]. Our crude rate was lower than the 30-day crude mortality rate from England (10.3 deaths/100,000 person-years) [11].

When we have both case fatality risks and mortality rates from population-based studies, we can compare the results for *E. coli* BSIs to other bacterial causes of BSIs to understand their relative burden of disease. The comparisons are meant to be illustrative in nature and should be viewed with caution since the studies are from different populations, cover different time periods, in some cases use different mortality definitions, and are from different bacterial pathogens, which could be associated with different BSI and patient characteristics (e.g., BSI source, comorbidities, course of treatment). Ideally, our comparisons would be drawn from multinational studies from similar areas using the 30-day mortality definition with rates directly age and sex standardized to the same standard population, which is an area for future research. The mortality rates for other causes of BSIs presented below were calculated by multiplying the reported case fatality risk by the incidence rate [5] if they were not reported in the manuscript. If the definition of mortality was in-hospital mortality, then the mortality rate is an underestimate of the true population mortality rate [25]. For *Staphylococcus aureus* BSIs, studies from Finland (2004–2007, 30-day), Calgary (Canada, 2000–2006, in-hospital), and Skaraborg (Sweden, 2003–2005, 30-day) identified case fatality risks of 16.9, 25 and 19.1%, respectively, and mortality rates of 3.5, 4.9 and 5.9 deaths/100,000 person-years, respectively [1, 39, 40]. For *Klebsiella* spp. BSIs, a study from Olmsted county (Minnesota, USA, 1998–2007) reported a 28-day case fatality risk of 14% with associated mortality rate of 1.6 deaths/100,000 person-years [41], and a study from western interior (BC, Canada, 04/01/2010–03/31/2017) identified a 30-day case fatality risk of 18% and a mortality rate of 2.2 deaths/100,000 person-years [42]. A study from Calgary (Alberta, Canada, 2000–2006) evaluating *Pseudomonas aeruginosa* BSIs reported an in-hospital case fatality risk of 29% with a mortality rate of 1.0 deaths/100,000 person-years [43]. For β-hemolytic streptococcal BSIs, a study from western interior (BC, Canada, 2011–2018) identified a 30-day case fatality risk of 11% and a mortality rate of 1.6 deaths/100,000 person-years [44]. If we view case fatality risk in isolation, our 30-day case fatality risk of 9.6% for *E. coli* BSIs is lower than the case fatality risks for other causes. Therefore, when using case fatality risk as the sole measure of disease severity [5], we might conclude that *E. coli* BSIs are less severe compared to the other bacterial causes of BSIs. However, if we compare our crude *E. coli* BSI mortality rate of 7.7 deaths/100,000 person-years to the other studies, we note that our mortality rate is the highest. *Escherichia coli* BSIs pose a significant burden of disease through mortality because they are much more common, which results in high incidence and mortality rates. This highlights the importance of exploring mortality through two lenses, rate and case fatality risk, which is only possible at a general population level when we use population-based study designs.

By presenting mortality through rates and case fatality risks, we had the ability to use different regression modelling approaches. Logistic regression is an approach for modelling mortality that is commonly reported in the literature [23, 27, 31, 33, 35, 36, 45–48] and by using this method we evaluated factors associated with the proportion of deaths or case fatality risk. Additionally, by using the Poisson regression model [23], we were able to explore factors associated with the number of 30-day deaths in *E. coli* BSIs adjusted for the underlying population-at-risk, which is the mortality rate. Therefore, we were able to gain insight into the factors impacting two measures of mortality and we found that the drivers for mortality rate and case fatality risk were similar. The explanatory variables that were associated with both the 30-day mortality rate and 30-day case fatality risk were region, age category, and sex although there was an interaction between age and sex in the model for 30-day case fatality risk. Additionally, location of onset, and 3GC resistance were associated with 30-day case fatality risk. The number of explanatory variables for the Poisson regression model were limited because population data were only available stratified by year, age and sex. Even in light of the data limitations affecting mortality rate modelling, it still provides important insight into burden of disease and can easily be added to the analyses of future population-based studies.

Hospital-onset and 3GC-R *E. coli* BSIs both significantly increased the odds of 30-day mortality. These findings are consistent with previous studies (Hospital-onset [6, 7, 14, 33], and 3GC-R [9, 31, 33, 45]). A meta-analysis from a 2014 systematic review also identified a significant increase in the risk of dying within 30-days of BSI diagnosis, when patients had 3GC-R *E. coli* BSIs compared to those with 3GC-S *E. coli* BSIs [49]. Interventions could be directed at decreasing 3GC-R or hospital-onset *E. coli* BSIs in order to reduce 30-day mortality in patients with *E. coli* BSIs.

The demographic factors, age and sex, are known to be important factors related to mortality. We found that *E. coli* BSI patients ≥70-years-old and those that were male experienced 30-day mortality at a higher rate, which is consistent with previous general BSI studies that found the rate of mortality in BSIs was significantly higher with increasing age and in male patients [1, 10]. The relationship between age category and 30-day case fatality depended on the sex being considered. We did not identify a previous study that reported an interaction between age and sex, however there are reports of higher case fatality risks with increasing age [6, 11, 33, 36] and in males [1, 11, 14, 33, 35]. The significant interaction between age and sex reinforces the importance of considering interaction effects during multivariable model building.

There were significant regional differences in 30-day mortality rate and 30-day case fatality risk. However, since region is a proxy for other unmeasured variables, we are unable to propose explanations for these regional differences. However, by including region as a fixed variable in the multivariable regression models, we were able to control for the regional differences while estimating the association between the other factors, and either 30-day mortality rate or case fatality. For future research projects, more detailed information regarding specific regional and population variables could be collected, which would facilitate exploration of the regional differences. During our 5-year study, we did not find significant changes over time in 30-day mortality rate or case fatality risk. When we compare results from a previous Finnish study (2004–2007) [1] to our results from Finland (2014–2018), we see 30-day case fatality risks in *E. coli* BSIs of 8 and 9.1%, and 30-day crude mortality rates of 3.5 deaths/100,000 person-years (calculated) and 8.2 deaths/100,000 person-years, respectively. There is a 13.8% increase in case fatality risk and a 133% increase in the mortality rate between the two studies. Considering the small increase in case fatality risk, the large increase in mortality rate can be mostly attributed to a substantial increase in the *E. coli* BSI incidence rate between the two studies. This comparison is only based on one area but we may have identified increases in the mortality rate over time, if our study covered a longer time period.

Our study did have some limitations that should be noted. We were only able to report community-onset and not further characterize these community-onset BSI episodes into healthcare-acquired and community-acquired. Detailed data were not available for co-morbidities, source of BSI, additional antimicrobial susceptibility results, treatment, length of hospital stay prior to positive culture, or other burden of disease outcomes (e.g., length of hospital stay, hospital costs, or measures of morbidity). We did not have information on culturing rates. The results from our study should only be generalized to other high-income countries [50]. One population-based study from Thailand [9], an upper-middle-income country [50], presented *E. coli* BSI mortality data, but more population-based research in low, lower-middle, and upper-middle-income countries is required to understand from a global perspective, the contribution of mortality to the burden of disease from *E. coli* BSIs.

## Conclusions

Our multinational population-based study identified that *E. coli* BSIs have a considerable burden of disease from 30-day mortality. Region, age, and sex were associated with both 30-day mortality rate and 30-day case fatality risk; in addition, 3GC resistance and location of onset were associated with 30-day case fatality risk. Because *E. coli* BSIs are much more common than other bacterial causes of BSIs, our population-based 30-day mortality rate was higher than that reported for other bacterial causes of BSIs, including *Staphylococcus aureus*, *Klebsiella* spp., β-hemolytic streptococci, and *Pseudomonas aeruginosa*. Our study highlights the importance of using population-based mortality data to evaluate mortality rate in addition to case fatality risk because they provide insights into different aspects of mortality. Even though *E. coli* BSIs have lower case fatality than some other bacterial causes of BSI and therefore are sometimes viewed as less severe, since *E. coli* is the most common cause of BSIs, it has a very substantial impact on human health as a result of mortality.

## Supplementary Information

**Additional file 1: Table S1.** Proportion of *E. coli* bloodstream infection patients with 30-day mortality by region that were resistant to third-generation cephalosporins and location of onset. **Table S2.** Counts of 30-day mortality and incident *E. coli* bloodstream infections, length of patient follow-up, case fatality risks, and crude mortality rates. **Table S3.** Crude odds ratios for the univariable logistic regression models estimating associations between 30-day mortality in *E. coli* bloodstream infections, and region, year, location of onset, third-generation cephalosporin resistance, sex and age. **Table S4.** Directly age and sex standardized *E. coli* bloodstream infection mortality rates for overall, third-generation cephalosporin-resistant and susceptible *E. coli* bloodstream infections. **Table S5.** Crude incidence rate ratios for the univariable Poisson regression models estimating associations between *E. coli* bloodstream infection 30-day mortality rates, and region, year, sex and age.

## Data Availability

The aggregated datasets analyzed during the current study may be available from the corresponding author on reasonable request.

## Abbreviations

3GC: Third-generation cephalosporin
3GC-R: Third-generation cephalosporin-resistant
3GC-S: Third-generation cephalosporin-susceptible
BSI: Bloodstream infection
BSIs: Bloodstream infections
CI: Confidence interval
*E. coli*: *Escherichia coli*
ESBL: Extended-spectrum β-lactamase
EU28: European Union 28-country
IRR: Incidence rate ratio
OR: Odds ratio
