# Supplementary material for: Mortality in Escherichia coli bloodstream infections: a multinational population-based cohort study
Source: BMC Infect Dis. 2021 Jun 25;21:606. doi: 10.1186/s12879-021-06326-x (PMC8229717; doi:10.1186/s12879-021-06326-x)
Supplement: Supplementary file 1 — Additional file 1: Table S1. Proportion of E. coli bloodstream infection patients with 30-day mortality by region that were resistant to third-generation cephalosporins and location of onset. Table S2. Counts of 30-day mortality and incident E. coli bloodstream infections, length of patient follow-up, case fatality risks, and crude mortality rates. Table S3. Crude odds ratios for the univariable logistic regression models estimating associations between 30-day mortality in E. coli bloodstream infections, and region, year, location of onset, third-generation cephalosporin resistance, sex and age. Table S4. Directly age and sex standardized E. coli bloodstream infection mortality rates for overall, third-generation cephalosporin-resistant and susceptible E. coli bloodstream infections. Table S5. Crude incidence rate ratios for the univariable Poisson regression models estimating associations between E. coli bloodstream infection 30-day mortality rates, and region, year, sex and age. [file 12879_2021_6326_MOESM1_ESM.pdf]

Additional File 1

Contains: Supplementary Tables 1 – 5

For manuscript: Mortality in *Escherichia coli* bloodstream infections: a multinational population-based cohort study

Authors: Melissa C MacKinnon[1], Scott A McEwen[1], David L. Pearl[1], Outi Lyytikäinen[2], Gunnar Jacobsson[3,4], Peter Collignon[5,6], Daniel B. Gregson[7,8], Louis Valiquette[9], and Kevin B Laupland[10,11,12]

Institutional Affiliations:
[1]–Department of Population Medicine, University of Guelph, Guelph, Ontario, Canada
[2] –Department of Health Security, National Institute for Health and Welfare, Helsinki, Finland
[3]–Department of Infectious Diseases, Skaraborg Hospital, Skövde, Sweden
[4]–CARe - Center for Antibiotic Resistance Research, Institute of Biomedicine, University of Gothenburg, Gothenburg, Sweden
[5]– Department of Infectious Disease and Microbiology, The Canberra Hospital, Garran, Australian Capital Territory, Australia
[6]– Medical School, Australian National University, Acton, Australian Capital Territory, Australia
[7]–Departments of Medicine, and Pathology and Laboratory Medicine, University of Calgary, Calgary, Alberta, Canada
[8]–Alberta Health Services, Calgary Zone, Calgary, Alberta, Canada
[9]–Department of Microbiology-Infectious Diseases, Université de Sherbrooke, Sherbrooke, QC, Canada
[10]–Department of Medicine, Royal Inland Hospital, Kamloops, British Columbia, Canada
[11]–Department of Intensive Care Medicine, Royal Brisbane and Women's Hospital, Brisbane, Queensland, Australia
[12]–Faculty of Health, Queensland University of Technology, Brisbane, Queensland, Australia

Corresponding Author

Melissa C. MacKinnon
melissa.c.mackinnon@gmail.com

<u>Supplementary Table 1</u> – Proportion of *E. coli* bloodstream infection patients with 30-day mortality by region that were resistant to third-generation cephalosporins and location of onset based on data from the multinational population-based cohort study (2014 to 2018).

**Region**	**Proportion resistant to 3GC (#R/Total)**	**Proportion community-onset (Community-onset/Total)**	**Proportion hospital-onset (Hospital-onset/Total)**
Calgary	24.2% (109/450)	71.3% (321/450)	28.7% (129/450)
Finland	9.3% (210/2252)	65.4% (1472/2252)	34.6% (780/2252)
Sherbrooke	4.7% (2/43)	69.8% (30/43)	30.2% (13/43)
Skaraborg	3.5% (5/142)	78.2% (111/142)	21.8% (31/142)
Western interior	13.5% (10/74)	71.6% (53/74)	28.4% (21/74)
Total	11.4% (336/2961)	67.1% (1987/2961)	32.9% (974/2961)

Abbreviations: 3GC – Third-generation cephalosporins; R – Resistant

Supplementary Table 2 – Counts of 30-day mortality and incident *E. coli* bloodstream infections, length of patient follow-up, case fatality risks, and crude mortality rates based on data from a multinational population-based cohort study (2014 to 2018).

**Region**	**Count of 30-day mortality in *E. coli* BSI cases**	**Count of incident *E. coli* BSI cases**	**Length of follow-up (patient-years)**	**Case fatality risk (%)**	**Crude mortality rate (deaths/100,000 person-years)**
Calgary	450	3773	8185926	11.9	5.5
Finland	2252	24629	27426758	9.1	8.2
Sherbrooke	43	596	820430	7.2	5.2
Skaraborg	142	1347	1319420	10.5	10.8
Western interior	74	578	924714	12.8	8.0
Total	2961	30923	38677248	9.6	7.7

Abbreviation: BSI – Bloodstream infection

Supplementary Table 3 – Crude odds ratios (95% confidence intervals and p-values) for the univariable logistic regression models estimating associations between 30-day mortality in *E. coli* bloodstream infections, and region, year, location of onset, third-generation cephalosporin resistance, sex and age based on data from a multinational population-based cohort study (2014 to 2018).

Variable	OR	95% CI	p-value
**Region**			< 0.001[a]
Finland	1.00	referent	
Calgary	1.35	1.21-1.50	<0.001
Sherbrooke	0.77	0.56-1.06	0.107
Skaraborg	1.17	0.98-1.40	0.084
Western interior	1.46	1.14-1.87	0.003
**Year**			0.166[a]
2014	1.00	referent	
2015	0.90	0.80-1.02	0.097
2016	0.92	0.81-1.04	0.167
2017	0.87	0.77-0.98	0.020
2018	0.88	0.78-0.99	0.036
**Location of Onset**			
Community-onset	1.00	referent	
Hospital-onset	2.53	2.33-2.75	<0.001
**3GC-R**			
Susceptible	1.00	referent	
Resistant	1.63	1.44-1.84	<0.001
**Sex**			
Female	1.00	referent	
Male	1.49	1.38-1.61	<0.001
**Age Category**			
<70-years-old	1.00	referent	
≥70-years-old	1.80	1.65-1.96	<0.001

Abbreviations: OR – Odds ratio; CI – Confidence interval

[a] Overall p-value for variable estimated using a likelihood ratio test

Supplementary Table 4 – Directly age and sex standardized *E. coli* bloodstream infection mortality rates for overall, third-generation cephalosporin-resistant and susceptible *E. coli* bloodstream infections based on data from a multinational population-based cohort study (2014 to 2018).

**Variable**	**Directly[a] Standardized Mortality Rate[b] (Rank[c])**		
	**Overall**	**3GC-R**	**3GC-S**
**Region**			
Calgary	8.9 (2)	2.0 (1)	6.9 (4)
Finland	8.5 (3)	0.8 (3)	7.7 (2)
Sherbrooke	5.4 (5)	0.3 (4)	5.1 (5)
Skaraborg	9.6 (1)	0.3 (4)	9.2 (1)
Western interior	8.4 (4)	1.3 (2)	7.2 (3)
**Year**			
2014	8.7	1.0	7.7
2015	8.1	0.9	7.2
2016	8.4	1.0	7.4
2017	8.4	0.9	7.5
2018	8.9	0.9	7.9
**Total**			
Overall	8.5	1.0	7.6

Abbreviations: 3GC-R – Third-generation cephalosporin-resistant; 3GC-S – Third-generation cephalosporin-susceptible; CI – Confidence interval

[a] Standard population – EU28 2018 population
[b] Units for the rates are deaths/100,000 person-years
[c] Relative ranking of rates for regions from highest (1) to lowest (5)

<u>Supplementary Table 5</u> – Crude incidence rate ratios (95% confidence intervals and p-values) for the univariable Poisson regression models estimating associations between *E. coli* bloodstream infection 30-day mortality rates, and region, year, sex and age based on data from a multinational population-based cohort study (2014 to 2018).

**Variable**	**IRR**	**95% CI**	**p-value**
**Region**			<0.001[a]
Finland	1.00	referent	
Calgary	0.67	0.60-0.74	<0.001
Sherbrooke	0.64	0.47-0.86	0.004
Skaraborg	1.31	1.11-1.55	0.002
Western Interior	0.97	0.77-1.23	0.828
**Year**			0.254[a]
2014	1.00	referent	
2015	0.95	0.85-1.07	0.402
2016	1.01	0.90-1.13	0.874
2017	1.02	0.91-1.15	0.672
2018	1.08	0.97-1.21	0.157
**Sex**			
Female	1.00	referent	
Male	1.00	0.93-1.08	0.929
**Age Category**			
<70-years-old	1.00	referent	
≥70-years-old	19.99	18.41-21.70	<0.001

Abbreviations: IRR – Incidence rate ratio; CI – Confidence interval

[a] Overall p-value for variable estimated using a likelihood ratio test
